# Genetic engineering of novel yellow color african violet (*Saintpaulia ionantha*) produced by accumulation of Aureusidin 6-*O*-glucoside

**DOI:** 10.1186/s12575-022-00164-0

**Published:** 2022-02-09

**Authors:** Amir Rajabi, Leila Fahmideh, Mojtaba Keykhasaber, Valiollah Ghasemi Omran

**Affiliations:** 1 Department of Plant Breeding and Biotechnology, University of Zabol, 98613-35856 Zabol, Iran; 2 Department of Plant Breeding and Biotechnology, Gorgan University of Agricultural Sciences and Natural Resources, Gorgan, Iran; 3 Department of Plant Pathology, University of Zabol, Zabol, Iran; 4 Genetic and Agricultural Biotechnology Institute of Tabarestan, University of Agriculture Science and Natural Resources, Sari, Iran

**Keywords:** Anthocyanin, Flower color, Genetic engineering, Ornamental flowers, Pigment biosynthetic pathway

## Abstract

**Background:**

Flower color is one of the main characteristics of ornamental plants. Aurones are light yellow flavonoids produced in the petals of a limited number of plant species including snapdragon (*Antirrhinum majus*). As a commercially-recognized species, African violet can be found in various colors except yellow. This research, aiming at changing the petals’ color of African violet from white to yellow, was conducted using the simultaneous expressions of chalcone 4’-*O*-glucosyltransferase (*4’CGT*) and aureusidin synthase (*AS1*) genes without the need for silencing anthocyanin biosynthesis pathway genes via both transient and stable transfer methods.

**Results:**

The transient gene transfer among transgenic plants led to a clear change of petals’ color from white to light yellow. This occurs while no change was observed in non-transgenic (Wild type) petals. In total, 15 positive transgenic plants, produced via stable gene transfer, were detected. Moreover, since their flower color was yellow, both genes were present. Meanwhile, the corresponding transformation yield was determined 20-30%. The transformation, expression and integration of genes among T0 transgenic plants were verified using the PCR, qRT-PCR and Southern blotting techniques, respectively. Furthermore, the probable color change of petals’ cross-section and existence of Aureusidin 6-*O*-glucoside (*AOG*) compound were determined using a light microscope and HPLC-DAD-MSn analysis, correspondingly.

**Conclusions:**

Generally, the creation of aurones biosynthesis pathway is only viable through the simultaneous expression of genes which leads to color change of African violet’s petal from white to yellow. This conclusion can lead to an effective strategy to produce yellow color in ornamental plant species.

**Supplementary Information:**

The online version contains supplementary material available at 10.1186/s12575-022-00164-0.

## Background

Production of new ornamental cultivars with different colors of flowers is one of the main goals in floriculture [1]. Variability in flower color can increase the commercial value and marketing of ornamental plants [2]. The global use of ornamental plants is more than $300 billion and the area under cultivation in 2018 is estimated 2,600,000 and 4200 ha worldwide and Iran respectively [2, 3]. Pigments in higher plants are generally grouped in three major classes: flavonoids, carotenoids, and betalains [4]. Anthocyanins are a class of flavonoids responsible for a range of colors: from orange to red, violet and blue. Similarly, carotenoids and betalains mainly yield yellow or red colors. Flavonoids such as chalcone and flavone are pale-yellow and often invisible with human eye [4].

**Fig. 1:**
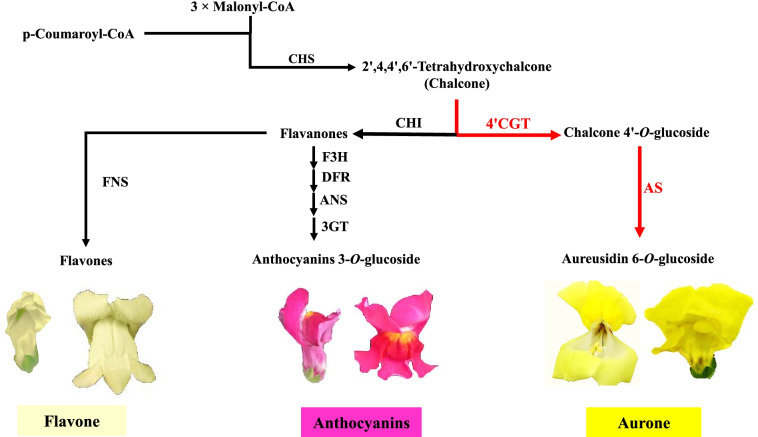
Simplified diagram of biosynthetic pathways flavonoids in *A. majus*. The 4’-*O*-glucosylation of chalcone by cytosolic *4’CGT* followed by oxidative cyclization by vacuolar *AS* is the biochemical basis of aurone 6*-O-*glucoside biosynthesis *in vivo*. Chalcone is the branching point of aurone pathway from the flavone/anthocyanin pathway. *CHS*, chalcone synthase; *CHI*, chalcone isomerase; *F3H*, flavanone 3-hydroxylase; *DFR*, dihydroflavonol 4-reductase; *ANS*; anthocyanidin synthase; *3GT*, anthocyanin 3-*O*-glucosyltransferase; *FNS*, flavone synthase; *AS*, aureusidin synthase; *4’CGT*, chalcone 4’-*O*-glucosyltransferase; THC, tetrahydroxychalcone; PHC, pentahydroxychalcone. Red arrows, the aurone biosynthetic pathway *in vivo* reported in this study

Aurone is a class of rare flavonoids with brighter yellow present flowers in a limited number of species, such as *A. majus*, *Cosmos bipinnatus* (garden cosmos), and *Limonium* (sea-lavender) [4–6]. Figure 1 shows flavonoids and a simple diagram of their biosynthetic pathways in *A. majus*. Biosynthesis of Aureusidin 6-*O-*glucoside (yellow pigment) and associated enzymes (*AS1* and *4’CGT*) have been well characterized in this plant [5]. 2’,4’,6’,4 tetrahydroxychalcone (THC) is the first compound in this pathway, which is the catalytic product of the key enzyme chalcone synthase (*CHS*). *THC* is glucosylated at its 4’-hydroxyl group in the cytoplasm, before transfer to the vacuole, and later converted to aureusidin 6-*O*-glucoside via the catalysis of *AS1* [5]. THC can later be converted to aurones, flavanones, and other classes of flavonoids, including anthocyanins.

African violet (*Saintpaulia ionantha* H. Wendl.; Gesneriaceae) is a commercial ornamental plant that can be easily propagated [7]. African violet has more than 22 species and 2000 cultivars with diverse petal color, floral shape, and color range of white, red, purple, and pink [8]. The *Saintpaulia* ‘*Jolly* Diamond’ cultivar has white petals; the number of petals is much more than its flowers and the petals hang slightly as they grow inwards [9]. Despite the variety of colors available among African violets, however, yellow has not yet been observed in this species [5, 7].

Transformation technology has potential to produce novel flower phenotypes that are not available in nature [1]. Genetic engineering can be used to create novel flower colors in a variety of ways: (a) by introducing new genes that encode enzymes to create new biosynthetic pathways that do not exist, (b) by directing biosynthetic pathways through up-regulation of existing genes, or (c) by suppressing biosynthetic pathways through down-regulation of genes [10]. However, genetic transformation is a valuable tool that can be exploited in plant functional genomics, gene discovery, and gene characterization [11].

Transient gene transformation (agroinfiltration system) using *Agrobacterium*-mediated Transformation (ATMT) in different plant tissues allows for rapid and scalable development of functional genomics assays [12–15]. This method is an efficient tool aimed at studying the function of the gene construct and tests it before stable transfer [16]. Stable transfer, on the other hand, is a lengthy process that often requires tissue culture techniques for full plant growth from transformed cells or tissues. In this method, T-DNA is artificially integrated into the genome of host cell using ATMT; therefore, it subsequently passed on to the next generation [17, 18]. Transgenic yellow flowers of *Torenia hybrida* are produced by co-expression of the *4’CGT* and *AS1* genes along with RNAi-mediated silencing of flavanone 3-hydroxylase (*F3H*) or dihydroflavonol 4-reductase (*DFR*) genes [5]. In transformed *Ipomoea nil* petals, the co-expression of the *4’CGT* and *AS1* genes induced expression of *AOG* and intensified the pale yellow color of the primary petals to visible yellow [19]. Genetically engineered production of yellow petals in important ornamental plants such as *Geranium*, *Lathyrus odoratus*, *Cyclamen* and *Saintpaulia* has not been reported [7, 19].

In spite of the presence of various colors among African violet ornamental plant cultivars, it lacks the yellow cultivar. Therefore, due to the popularity of the African violet ornamental plant, the production of this plant with a new yellow color is very valuable commercially and is of considerable importance in the market. Thus, the present study aimed to change the color of African violet flowers from white to new yellow color through genetic manipulation (using both transient and stable transformation) and the simultaneous expression of *4’CGT* and *AS1* genes involved in aurone biosynthetic pathway without any necessity for silencing of anthocyanin biosynthesis genes (*CHI*, *F3H* and *DFR*).

## Results

### Transient expression of ***4’CGT*** and ***AS1*** genes in African violet petals

**Fig. 2:**
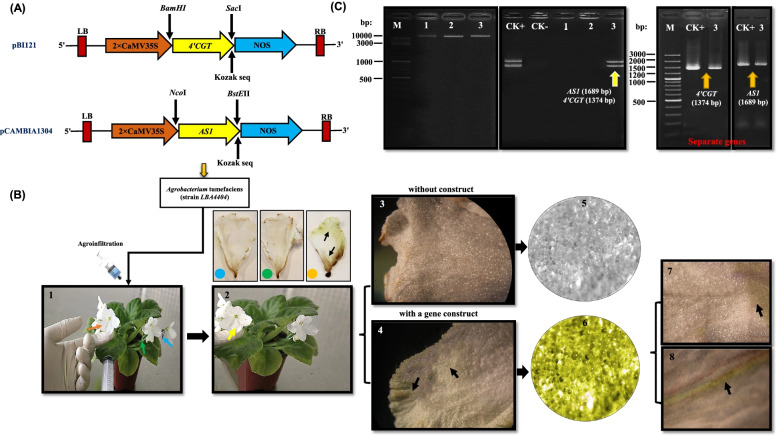
Restriction maps of transformation vectors and the results of *Agrobacterium* infiltration in African violet petals. **a** Binary vectors. The cloning vector pBI121 and pCAMBIA1304 containing the CaMV 35 S promoter driving the *4’CGT* and *AS1* genes respectively. **b** Agroinfiltration steps in African violet petals (1): The injection of *Agrobacterium* infiltration solution in the base of the petal by syringe without a needle, blue arrows without injection, a green arrow without gene construct and orange arrow with gene construct *4’CGT+AS1*; (2) a color change from white to yellow in petal influenced by gene construct *4’CGT+AS1*; (3, 4): Microscopes in the petal color injected samples with a gene construct and without construct; Light microscopes in the sample with a gene construct (5) and without a gene construct (6); (7): Shown are petal color on the adaxial side of sample injection with a gene construct; (8): Cross-sections arrow show that fluorescence is restricted to the pigmented adaxial epidermis of gene construct (Scalebar:100). **c** The results of extraction DNA and PCR detection of agroinfiltration petals; M: GeneRuler DNA Ladder Mix; CK-: Non-transgenic plants; CK+: cDNA of *A. majus* with mixture two primers *AS1* and *4’CGT*; (1): without injection; (2): injection without gene construct; (3): injection with gene constructs *4’CGT+AS1*

The function of *4’CGT* and *AS1* in the aurone biosynthesis was verified using the agroinfiltration method (Fig. 2b, c). Three days after infiltration, the color of the petals has changed from white to yellow (pale yellow), while no change was observed in the control group and the petals were injected with agroinfiltration solution without gene structure. Successful infiltration was later confirmed by light microscope. The results clearly showed the color change to yellow in surrounding parts injected with needle (Fig. 2b). Additionally, the presence both of *4’CGT*+*AS1* genes in infiltrated petals was confirmed by PCR analysis and these two genes were not detected in control plants (non-injected plant and injected plant without gene construct) (Fig. 2c). This experiment was repeated twice and similar results were obtained (data not shown).

### Transgenic Plants of African violet by simultaneous expression *4’CGT* and *AS1*

**Fig. 3:**
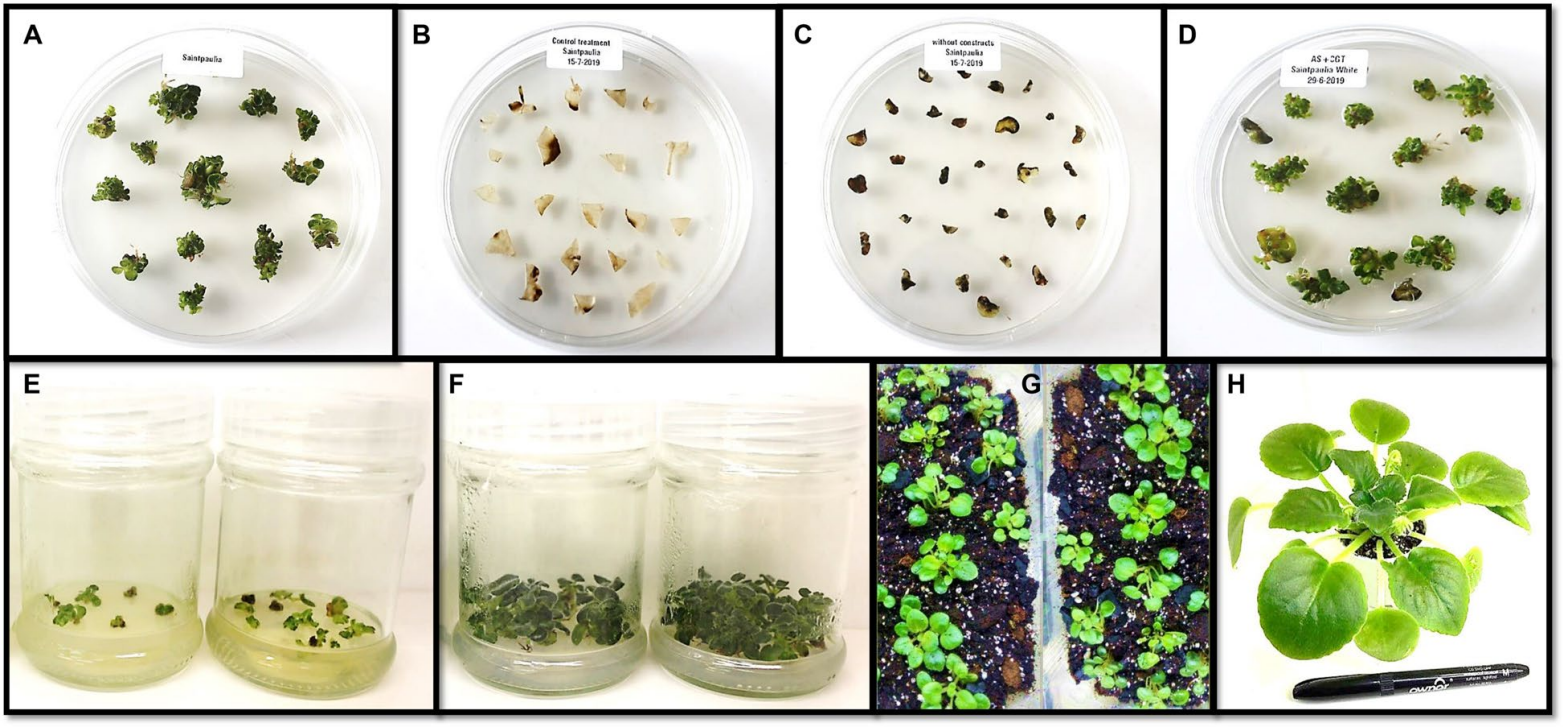
Generation process of transgenic plants African violet. **a** Plantlet regeneration in MS basal medium with 1 mg.L^−1^ 6-benzylaminopurine (BA) and 1 mg.L^−1^ indole-3-butyric acid (IBA). **b** Control of African violet leaves. **c** Control of African violet leaves without gene constructs. **d** *Agrobacterium*-mediated genetic transformation of African violet leaves with gene constructs. **e** Screening of *Agrobacterium*-mediated genetic transformation of leaves. **f** Induction of screened transforms. **g** Generation of transgenic seedlings. **h** Outdoor transplantation

**Fig. 4:**
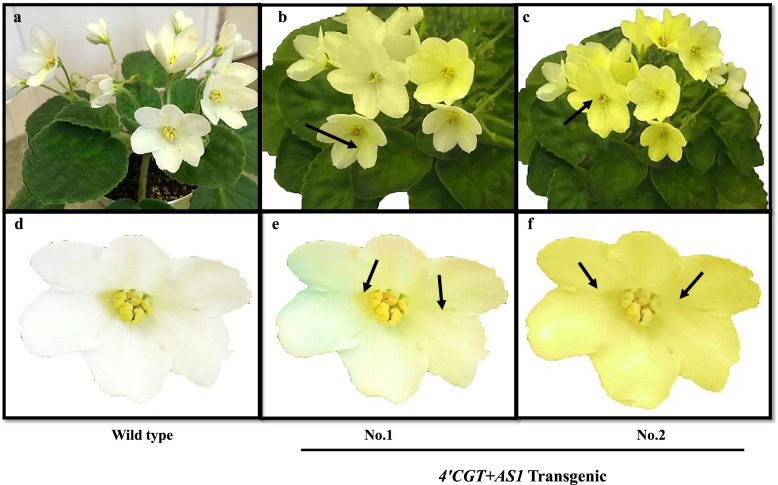
Alteration of flower color in transgenic African violet plants carrying *4’CGT+AS1* transgene. **a** Non-transgenic plant. **b** At the beginning of flowering, only a few flowers showed yellow regions on white petals. **c** All the flowers at the late-flowering stage in transgenic plants looked yellow. **d** Non-transgenic plant (Wild-type) petals. **e** to **f** Flower phenotypes from a spot of yellow color in the white background to complete yellow color observed in the same transgenic plant. The arrows showed a color change from white to yellow in different parts of *4’CGT+AS1* transgenic petals

To produce *AOG*, we inserted *4’CGT* and *AS1* genes into leaves through ATMT. Two binary vectors: pBI121 to express the *4’CGT* gene and pCAMBIA1304 to express the *AS1* gene were constructed. A few weeks after the transfermation of gene constructs, small white calli with green buds began to grow on some of the inoculated petiole explants. Totally, 15 putative independent transgenic plants were obtained from a total of 60 transformed explants (Table 1). 20–30% regeneration efficiency was achieved which enabled us to obtain 53 plantlets from 15 regenerated samples (Table 1; Fig. 3D). Regenerated plantlets were screened on Hygromycin and Kanamycin selection (Fig. 3E). Availability of Hygromycin and Kanamycin-resistant gene have successfully confirmed using PCR in putative transgenic plants. All putative transgenic plants acclimated and transferred to greenhouse until maturity (about 16 weeks). In transformants with white-yellow flowers, the area of yellow sectors increased in the white background of the petals during the late phase of flowering. With time, the color of most of the petals turned to yellow in all the late-born flowers of transgenic plants. In contrast, no change was observed in the petals of non-transgenic plants. No morphological difference other than the color change was observed between non-transgenic and transgenic plants (Fig. 4a-f).

**Table 1:** Analysis of plant transformation

No. Experiment	Total	Number of regenerations	Percentage of regeneration	Number of meristematic stems or plantlets	The average length of regenerated stems	Phenotype		Remarks
Transgenic African violet experiment 1	20	5	25%	17	5/4 cm		White-yellow	No seed
Transgenic African violet experiment 2	20	4	20%	15	7/5 cm		yellow	No seed
Transgenic African violet experiment 3	20	6	30%	21	7/0 cm		yellow	No seed
NT ( Wild type)	20	0	0	0	0		-	-

### Expression of ***4’CGT*** and ***AS1*** in transgenic African violet Flowers

**Fig. 5:**
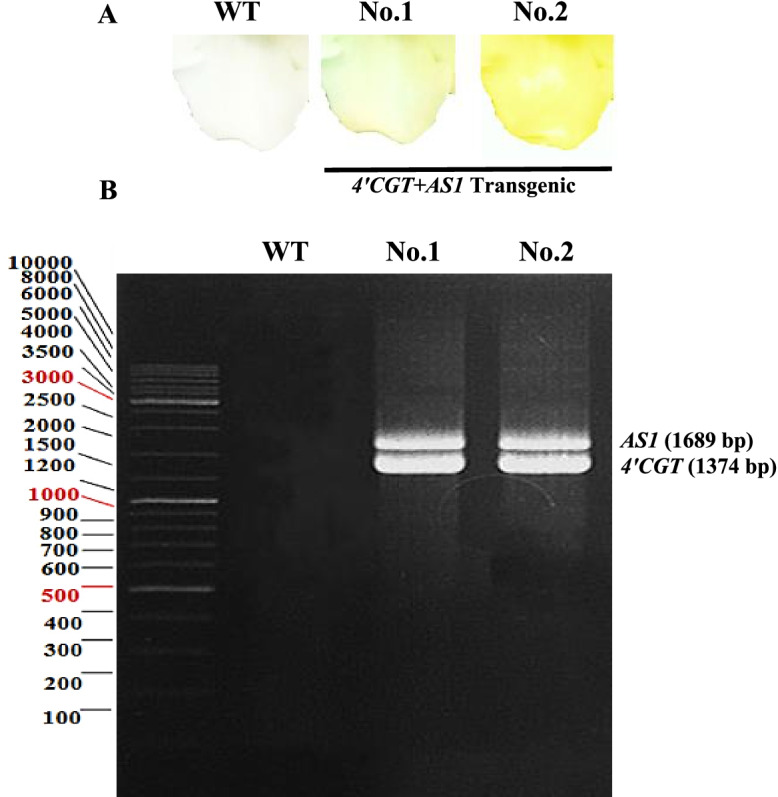
Results of PCR detection of wild-type and transgenic plants. **a** Extracted solutions from flowers of wild-type and transgenic petals. **b** WT: wild-type. No. 1, 2: Transgenic plants

We confirmed the expression of the transgenes with PCR. The size of PCR products was 1374 and 1689 bp for *4’CGT* and *AS1* genes, respectively (simultaneous expression *4’CGT* and *AS1* in T0 transgenic plants), identical with the positive control. These genes were not detected in non-transgenic plants (Fig. 5).

**Fig. 6:**
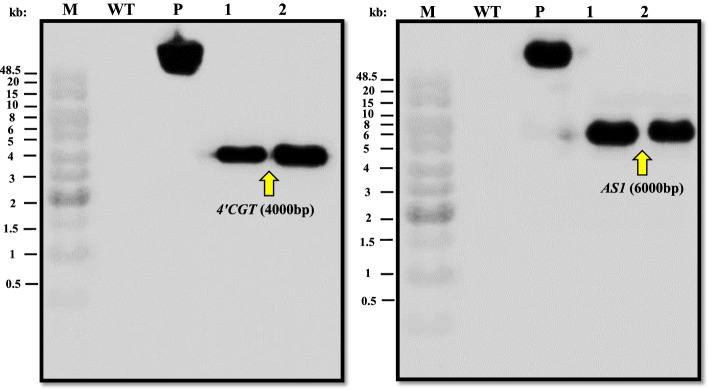
Results of southern blot hybridization of T0 generation transgenic plants. M: 1 kb ladder, Fermentas: P: pBI121-*4’CGT* and pCAMBIA304-*AS1* vectors, WT: Non-transgenic plants, 1, 2: T0 generation transgenic plants

Two samples from PCR positive transgenic plants (Fig. 5) were selected for further confirmation by Southern blot assay. For blotting, the researchers used specific 1374 and 1689 bp probes labeled with digoxin molecules. A Single signal was detected for each gene in the blotting of two transgenic African violet genomes, which were positive in PCR analysis, whereas no hybridization signal was observed in the non-transformed plant samples (African violet negative control). The lengths of hybridized fragments were 4000 and 6000 bp for *4’CGT* and *AS1* genes, respectively (Fig. 6).

### Evaluation of the expression pattern of ***4’CGT*** and ***AS1*** genes in transgenic petals

**Fig. 7:**
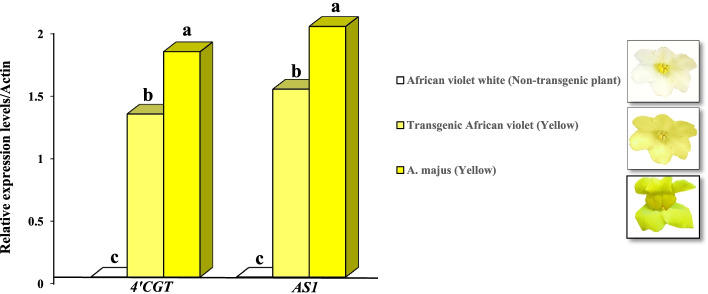
qRT-PCR confirmation of *4’CGT* and *AS1* transgenic plants. Relative expression levels/Actin of *4’CGT* and *AS1* genes in African violet white (Non-transgenic plant), Transgenic African violet (Yellow), and *A. majus* (Yellow). plants were investigated by qRT-PCR. Relative expression levels were normalized to a value of 1

The relative expression levels of *4’CGT* and *AS1* genes in transgenic plants with high expression levels were further confirmed by qRT-PCR. The amplification efficiency of genes ranged between 98.71% and 99.83% and correlation coefficients varied from 0.977 (*4’CGT*) to 0.996 (*AS1*) (Supplementary Table S1). These results indicated the expression of both genes studied in transgenic petals as compared to Non-transgenic petals. The results showed that the expression level of the studied genes was not naturally present in African violet white (Non-transgenic plant), while in transgenic African violets (yellow), it expressed significantly and was to some extent equal to *A. majus* (yellow) (Fig. 7).

### Microscopic analysis of transgenic petals

**Fig. 8:**
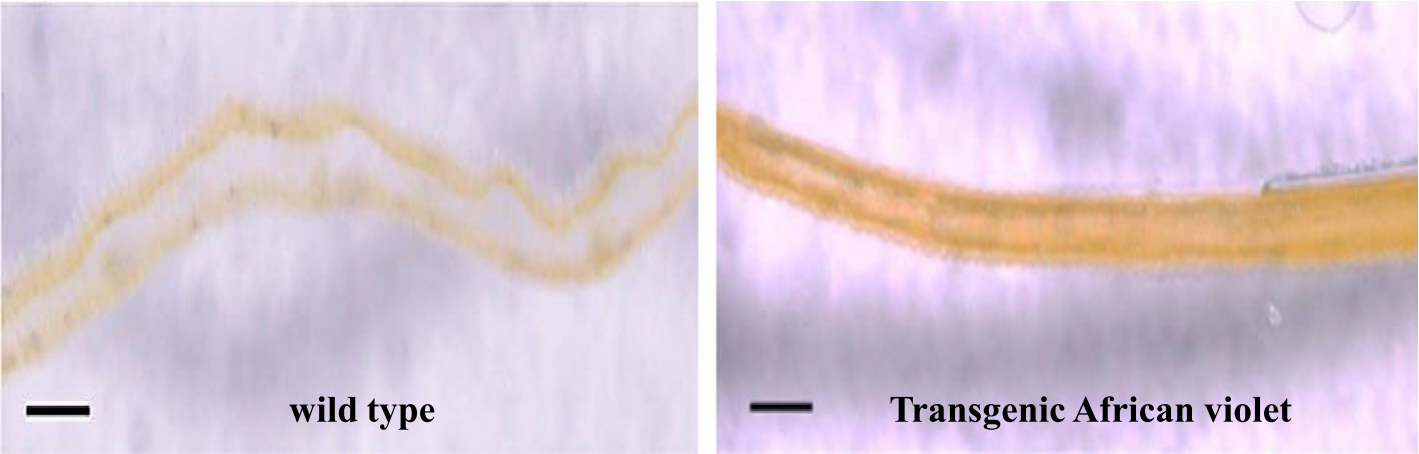
Light microscope observation of transverse section through petals of Transgenic African violet and wild type. Bars= 100 μm

To further study the expression of *4’CGT*+*AS1* genes in petals of transgenic plants, transverse sections of petals were examined by microscopic analysis (Fig. 8). In wild type, pale-yellow pigments (Chalcones) were localized in the adaxial epidermis (L1 layer) and the underlying mesophyll (L2 layer) appeared to be white. In contrast, both L1 and L2 layers were yellow in petals of transgenic plant (Fig. 8). It generally represents the merger of both genes together in yellow transgenic African violet.

### Aureusidin 6-***O*** glucoside accumulation in the ***4’CGT*** and ***AS1*** expressing transgenic petals

**Fig. 9:**
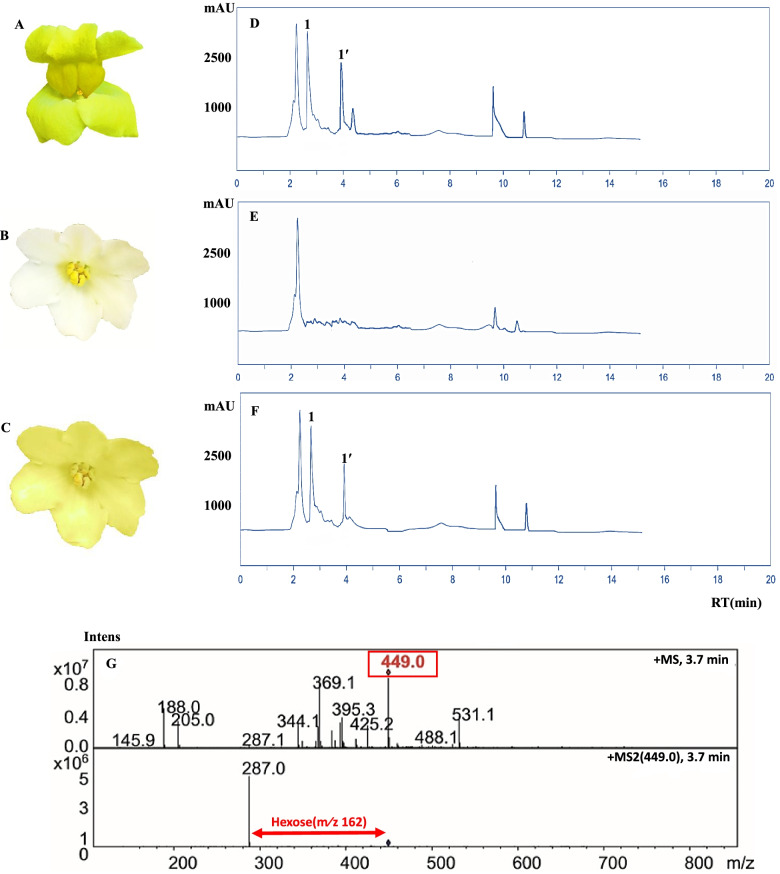
Aurone formation in African violet transgenic. **a** Flower of untransformed *A. majus*. **b** untransformed African violet. **c** transgenic African violet. **d** HPLC chromatogram of *A. majus* flowers. **e** untransformed African violet. **f** transgenic African violet. showing a eluting as peaks 1 and 1ʹ at 400 nm. Mass spectra and MS-MS fragmentations of m⁄z 449 of *AOG* from *A. majus*. **g** mAU, milliabsorbance units

Flavonoids were extracted from petals of not-transformed African violet, transformed African violet, and *A. majus.* These metabolites were then analyzed by HPLC. The HPLC chromatogram demonstrated that the yellow flowers of *A. majus* contained a compound that was not present in non-transgenic African violet (Fig. 9e) but was observed in transgenic African violet petals (Fig. 9f). Retention times (RT) in transgenic African violet and A. majus flowers were identical, thereby confirming the presence of *AOG* (Fig. 9a; Table 2). The amount of *AOG* component (Peak 1ʹ, RT 3.9 min) in flowers of the transgenic African violet and *A. majus* was 2.95 and 3.45 mg/g, respectively (Table 2). HPLC chromatogram showed that the components were properly separated. MS⁄MS analysis of peak 1ʹ, which exhibited an [M + H]- ion at m⁄z 449, yielded MS2 fragmentation at m⁄z 287 due to the loss of 162 atomic mass units (amu), corresponding to one glucose moiety (Fig. 9 g). Peak 1 is unlikely due to the saturation of peak 1ʹ as the two peaks are well separated and not tailing. In *A. majus*, a trace amount of molecular ion m⁄z 465 was revealed to be co-eluted with broad peak 1ʹ, which was fragmented at m⁄z 287 (data not shown) and tentatively identified as bracteatin-6-*O-*glucoside. Additionally, the mass spectra and UV⁄V are features of the peaks 1 and 1ʹ, a compound identified in *A. majus* and transgenic African violet (Fig. 9d, f) but not in the wild type (Fig. 9e). These features possibly correspond to a structural isomer of *AOG*.

**Table 2:** Flavonoid analysis of transgenic and non-transgenic flowers

		Flavonoid content, mg/g fresh petal weight				
(No)	Genotype	Aurone at 400 nm		Anthocyanidins at 520 nm	Flavones at 360 nm (NC)	Phenotype
		Peak 1 (2.5 min)	Peak 1′(3.9 min)			
NT	S. *Jolly Diamond*	UD	UD	UD	0.986	White
Transgenic African violet	*4′CGT*+*AS1*	0.528	2.95	UD	1.23	Yellow
*A. majus*	cv. Snap Yellow (Endogenous *Am4′CGT* and *AmAS1*)	0.632	3.45	UD	1.5	Yellow

UD, undetectable

Flavonoids in all the transgenic lines and nontransformants were identified by HPLC, and the data were summarized. Tentative peak identification: Peaks 1 and 1′, *AOG*; Peak 2,3,4, Anthocyanidins: Peak 5,6,7, Flavones (NC: naringenin chalcone and derivatives(

## Discussion

Genetic engineering supports the idea of changing as well as creating new colors in ornamental plants. This research has been conducted with the purpose of changing flower colors and creating new ones in African violet species. Flower and ornamental plants industry has been trying to develop new cultivars with specific characteristics such as new colors [20]. The development of ornamental cultivars with new flower colors, considering the major purpose of flower and ornamental plants industry, will result in increased economic value [21]. Ornamental cultivars of *Petunia*, *chrysanthemums*, *Rosa hybrid*, *Ipomoea nil*, *Rosa rugose* and *Nierembergia* species with respective colors of light pink, blue-violet, purple, light yellow, yellow and light violet have been produced using the simultaneous expression or overexpression of the corresponding genes [19, 22–25]. The anthocyanins were the principal pigments leading to the development of new colors including red, pink and blue. This is while aurone compound has been used to develop yellow-colored flowers in recent studies [19, 26].

Although African violet can be found in various colors, no color change has been reported through genetic engineering of this precious species. In this investigation, the white-colored petals of *S. ‘Jolly Diamond’* cultivar have been used. Therefore, no *CHI* is available to turn chalcone into *AOG*. Chalcone, considered as a key enzyme in flavonoid biosynthesis of flowering plants including aurones, is turned into colorless naringenin and plays an important role in color of ornamental flowers [2, 21]. In most plant species, chalcone is not the final product. Combined with other enzymes, it turns into other classes of flavonoids such as aurones, flavonones, dehydroflavonols and finally anthocyanins as a major class of colors [27]. In a molecular analysis investigation, two separate genes of chalcone synthesis (i.e., *SaCHSA* and *SaCHSD*) were detected in African violet [28]. It should be noted that the identification of chalcone in this ornamental plant species is highly significant to conduct this research.

For the first time, the simultaneous transfer of *AS1* and *4’CGT* genes was successfully conducted in African violet using *Agrobacterium tumefaciens* strain LBA4404 with respective plasmids of pCAMBIA1304 and pBI121. In an investigation carried out on African violet by [29], two strains including A218 and EHA105 were employed. The results indicated that only strain A281 led to more efficient and stable transfer of traits in leafstalk microsamples while strain EHA105 caused no regeneration. In another research by [30], *Agrobacterium tumefaciens* strains LBA4404 and EHA101 were used in *Pink Veil* cultivar of the African violet. It was found that strain LBA4404 (pTOK233) had better performance. According to this research and the investigation conducted by Kushikawa et al. [30], LBA4404 can be considered an appropriate strain in transfer of genes to African violet.

The transient gene transfer enables investigating the genetic construct. Besides, the flower color change can be evaluated without the need for tissue culture. The transgenic plants can also be selected at lower cost and time if this technique is applied [12, 31, 32]. This method, showing high transfer yield in a wide range of plant species, has been employed to study the African violet petals. To this end, agrobacterium containing gene construct has been injected into the base of petals. Three days later, the phenotypic evaluation of color change of petals as well as the existence of *4’CGT* and *AS1* genes in the transgenic petals were verified using light microscope and PCR test. According to the results, the *S. ‘Jolly Diamond’* cultivar, having white petals, is suitable for genetic modification and transfer of genes involved in the aurones biosynthesis pathway with the purpose of changing the flower color to yellow. Other researchers have also used the transient gene transfer method. Nazari et al [33] used this method to transfer monogenic (*Viola-F3ʹ5ʹH* gene) and constructs (*Viola*-*F3ʹ5ʹH* gene and *Iris*-DFR gene). After injection of gene constructs, they conducted to produce delphinine anthocyanin in petals of *Gerbera* flower (up to 44 and 75%, correspondingly), resulted in color change of petals into blue. In a similar research, the transfer of gene construct containing *F3ʹ5ʹH* and DFR gene from respective *Petunia hybrida* and *Iris* to the petals of *Gerbera jamesonii* caused a change in the level of anthocyanins among the injected petals and turned the stigma and pollen into blue [34]. Shang et al [35] investigated the effect of RNAi agroinflitration of chalcone synthase gene on the *A. majus* petals. By silencing this gene, no pigments were found in *A. majus* petals and their color changed from purple to white. The silence of chalcone isomerase gene involved in the biosynthesis pathway of *Petunia hybrida*’s pigments production led to the accumualation of anthocyanin while a decrease was observed in the endogenous mRNAs of the respective targets in the petals’ infiltrated zones of four colors of this plant species [36]. The production of transgenic plants is an expensive and time-consuming process. According to the literature, transient transfer is a fast, simple and economic method to test and activate constructs prior to their mass production as gene constructs are most likely transferred following their verification.

In the stable gene transfer method, a certain part of DNA containing a new gene or a combination of multiple genes is artificially inserted into an organism’s genome using laboratory methods [37]. Due to the complex nature of flavonoid biosynthesis pathway, the mere transfer of a gene to the plants might not have any specific effects on the biosynthesis pathways of flower’s pigments. Therefore, the genetic engineering of flower color has been investigated via transferring of multiple genes [38]. In such research, the white-colored petals of African violet were turned into yellow using two vectors (pBI121 and pCAMBIA1304 vectors for *4’CGT* and *AS1* gene expressions, respectively) without any need for silencing anthocyanin biosynthesis pathway genes (*CHI*, *DFR* and *F3H*). The results of this research are similar to those of To and Wang [26]. In that research, the *CHI* and *DFR* genes corresponding to *Petunia*’ cDNA pattern were cloned in pBI121 and pCAMBIA1304 carriers, respectively. By transferring them to *tobacco* via agrobacterium, different color patterns were developed compared to non-transgenic *tobacco*.

For the first time, the relative expression of both genes transferred to the petals was observed and verified in this research. While no analyses (qRT-PCR, Southern blotting and light microscope) were carried out to verify the results observed in previous investigations, the above-mentioned analyses have been conducted to demonstrate the transfer and integration of genes in genetically-modified plants. According to the qRT- PCR analysis, *4’CGT* and *AS1* genes were successfully expressed in the African violet petals. This is while non-transgenic African violet plants lack these two genes. To verify the integration process of the transferred genes to the genome of genetically-modified plants as well as determining the number of gene transcripts, the Southern blotting test was applied. Findings showed that the genetically-modified plants possessed one or two transcripts of the transferred genes. By investigating the genetically-modified plants having *4’CGT* and *AS1* genes sequencing, the band lengths were determined 4500 and 6000 bp, respectively. In a recent research, HPLC analysis was used to verify and quantify these genes in the transgenic *Ipomoea nil* ornamental plant [19].

In another investigation, the *4’CGT* and *AS1* genes were separately transferred from *A. majus* to the blue-colored petals of *Petunia*. According to the results, 4 out of 9 transgenic *SRY4’CGT* plants developed blue-white flowers. Moreover, the proportion of white-colored sectors gradually increased until the petal color turned completely white. In contrast, 3 out of 13 transgenic *SRYAS1* developed blue-white sectors. However, the amount of transgenic flowers did not increase and no complete white flowers were observed. Besides, the chalcone synthase significantly increased in the blue-colored sector while a decrease was observed in the white-colored Sect. [39]. In the blue-colored petals of *Torenia hybrida* ornamental plant, expression of these genes along with silencing *DFR* or *F3H* gene led to the development of yellow color. In an investigation by the RNAi technique, no change was reported in the appearance of flower [5]. Recently, the transfer of these genes to the petals of *Ipomoea nil* ornamental plant changed the color of flower from light yellow to yellow. However, most transgenic plants contained light brown-colored unbloomed flowers which might have consisted of necrotic cells [19]. In contrast, this research led to the production of 15 yellow-colored transgenic plants out of 60 explants transferred by ATMT. Meanwhile, there was no need for gene silencing (RNAi) and no other morphological difference was observed between transgenic and non-transgenic plants.

The flowers of yellow-colored *A. majus* cultivar produce a considerable amount of *AOG* and bracteatin 6-*O*-glucoside along with smaller quantities of the 4’-glucosides of THC and PHC [40, 41]. According to the HPLC analysis conducted on transgenic *Torenia hybrida* ornamental plant, the *AOG* compound was produced in the transgenic plants (0.422 mg/g) while there was no trace of such compound in non-transgenic [5]. The enzymatic formation of aurones observed in the yellow-colored *A. majus* flowers extracts indicated that the main pigments pathway to produce *AOG* was open followed by reduction in PHC-glucoside, bractatin-6 glucoside, THC-4 glucoside, respectively. In this research, the HPLC-DAD-MS chromatogram revealed the existence of *AOG* compound in the transgenic African violet plants containing yellow-colored petals. This is while no such compound is produced in the white-colored petals of non-transgenic African violet plants naturally.

## Conclusions

For the first time, the genetic engineering of aurone pigment biosynthesis pathway led to the production of yellow color in the white-colored African violet petals. This method was employed by simultaneous expression of *4’CGT* and *AS1* genes. Furthermore, agroinfiltration system was highly effective to evaluate the performance of genes and color of flowers. In this research, the *S. ‘Jolly Diamond’* cultivar containing white-colored petals was used. The simultaneous expression of *4’CGT* and *AS1* genes led to the formation of new yellow color. This is while the existence such phenotype was not reported in the African violet. The results of this investigation indicated that the simultaneous expression of these genes in the white-colored petals, containing chalcone, contributed to the accumulation of *AOG* as the final compound of aurones. As the African violet petals produce chalcone, the existence of malonyltransferase caused accumulation of aurones. The phenotypic and molecular evaluation of integration indicated that these genes, detected in the genome of transgenic petals, were not in a single locus. This observation also demonstrated the production of transgenics by clonal reproduction. Furthermore, yellow pigments and accumulation of *AOG* were observed in the petals of *4’CGT/AS1* transgenic plants while no change was detected in the petals of wild type. Therefore, transferring these genes to other ornamental plants can lead to the production of yellow-colored petals provided that white-colored petals contain chalcone to produce *AOG* inside the vacuoles. Moreover, the simultaneous expression of *4’CGT* and *AS1* was conducted without the need for silencing genes to reduce the anthocyanin biosynthesis adjustment. Thus, the mere application of these genes is applicable to change the petals’ color from white to yellow. Besides, this method can be considered an appropriate strategy to change the color of white petals as well as producing new color in the flowers of ornamental plant species.

## Materials and methods

### Vectors Construction

To clone the full-length cDNA of *4’CGT* and *AS1* genes, RNA from yellow petals of *A*. *majus* was isolated by TRIzol protocol as previously described [42]. Specific primer sets P1 and P2 (Supplementary Table S2) were used to amplify full-length reading frames of *4’CGT* (1374 bp) and *AS1* (1689 bp), respectively. PCR products were then analyzed by 1% agarose gel electrophoresis. After purification, PCR products were cloned into Pet28a Easy vector (Promega) and then sequenced by an automatic DNA sequencer (Sanger Sequencing method, ABI 3730 XL). The full-length cDNA of the *4’CGT* gene was cloned into the pBI121 binary vector using *SacI* and *BamHI* restriction sites. *AS1* gene was cloned into pCAMBIA1304 vector by *NcoI* and *BstEII* restriction sites, respectively (Supplementary Table S2; Fig. 2a). All these vectors were verified by restriction enzyme cutting and sequencing.

### *Agrobacterium*-Mediated Transformation

*Agrobacterium tumefaciens* (strain *LBA4404*) provided from of the Institute ABRII (Agricultural Biotechnology Research Institute of Iran) was used to carry recombinant binary vectors and *Agrobacterium*-mediated plant transformation assay. For transformation, the researchers used the electroporation (BTX™ ECM™ 630, USA) at 25 µF, 400 Ω, and 1.25 kV. 1 ml of LB medium was then added immediately into the mixture, incubated at 28˚C for 3 h. 100 µl of the following bacterial mixture was transferred onto selective LB medium (containing 50 mg.L^−1^ Kanamycin and 100 mg.L^−1^ Rifampin) as described in the manufacture’s protocol.

## Plant materials and growth conditions

African violet (*S. Jolly Diamond*), was used in the present study. All *Saintpaulia* genotypes were provided from the genotype collection of the Institute GABIT (Genetics and Agricultural Biotechnology Institute of Tabarestan) and the plant material was maintained in greenhouses under controlled conditions. For plant transformation, the leaves were sterilized with 1% sodium hypochlorite for 20 min, washed thoroughly with sterile water, and cultured to germinate on MS basal medium (pH 5.7) containing MS salts [43] 2% sucrose, 0.8% bacto-agar, 1 mg.L^−1^ 6-benzyl amino purine (BA), and 1 mg.L^−1^ indole-3-butyric acid (IBA). The cultures were then incubated in a growth chamber under the condition of 25±1ºC, a 16-h florescent light (100 µmol/m2/sec), and a 8-h dark cycle. 8 weeks later, young leaves were collected and used for the transformation test.

### Transient expression via infiltration of petal

African violet flowers were infiltrated with *Agrobacterium tumefactions* strain LBA4404 harboring both pBI121 and pCAMBIA1304. For this, the LBA4404 cells were cultured in 10 ml LB broth with antibiotics overnight, pelleted and re-suspended in a medium #1003 (AB media salts + NaH2PO4 240 mg.L^−1^ + glucose 10 g/l + MES 14.693 g/l) supplemented with 100 µM acetosyringone and cultured for 4 h [35]. The cells were then pelleted and re-suspended to a concentration of A600 = 0.5 in 1% (w/v) glucose solution (PH 5.3) supplemented with 100 µM acetosyringone [35]. Flower buds or opened flowers were pierced with a needle and infiltrated with the *Agrobacterium* culture using a syringe. Three days after injection and subsequent change in specimen’s conditions, the injected petals (both with and without gene construct) were separated from non-injected ones. After DNA extraction, the existence of two genetic parts was examined using PCR test. To further investigate the color change of petals, the cross section of the injected petals (with and without gene construct) was evaluated using a light microscope.

### Stable transformation

A single colony of plasmid-carrying *Agrobacterium* was picked up and incubated in 10 ml LB medium containing 50 mg.L^−1^ Kanamycin (Km) and 100 mg.L^−1^ Rifampin (Rif) at 28˚C overnight. Bacteria cells (OD 600 - 0.5 - 0.6) were then washed and resuspended in AB medium (5 g/l glucose; 1 g/l NH_4_Cl; 0.3 g/l MgSO_4_∙7H_2_O; 0.15 g/l KCl; 10 mg.L^−1^ CaCl2; 2.5 mg.L^−1^ FeSO_4_∙7H_2_O; 3 g/l K_2_HPO_4_; 1.15 g/l NaH_2_PO_4_∙H_2_O) without antibiotics. The cultures were incubated at 28 °C for 6 h to reach mid-log phase, followed by the addition of 100 µl of acetosyringone (AS; SigmaAldrich, St. Louis, MO, USA) and further incubation at 28 °C for 4 h. The bacterial suspension was then centrifuged at 3000 rpm for 10 min, and the pellet was dissolved in MS medium (MS salts; 0.9 mg.L^−1^ thiamine; 1 mg.L^−1^ BA; 1 mg.L^−1^ IBA; 200 mg.L^−1^ KH_2_PO_4_; pH 5.6) supplemented with 100 µM acetosyringone and 5% glucose. The suspension was then diluted to final OD600 0.6-0.8. One day before the infection, leaves were excised from 4- and 6-week-old *in vitro* grown African violet and incubated on MS solid medium supplemented with 100 µM acetosyringone. Infection was carried out by adding 15 ml of diluted *Agrobacterium* suspension to the pre-cultured explants for 1 h. Excess *Agrobacterium* explants were blotted on sterile filter papers to remove excess liquid. The infected explants were transferred on MS solid medium containing 100 µM acetosyringone and 5% glucose, and left to grow for 3 days in a dark condition at 28˚C. Then they were washed 2-3 times with shaking (the first shaking at 220 rpm and subsequent shakings at 100 rpm; 30 min each time) in MS liquid medium with 500 mg.L^−1^ cefotaxime. Explants were dried with sterile filter papers and transferred to MS medium (MS salts; Nitsch vitamins; 1% sucrose; 1 mg.L^−1^ BA; 1 mg.L^−1^ IBA 0.7% bacto-agar; pH 5.8) with 250 mg.L^−1^ cefotaxime (for inhibition of *Agrobacterium* growth), 50 mg.L^−1^ Kanamycin (for selection of pBI121/*4’CGT* vector) and 75 mg.L^−1^ Hygromycin (for selection of pCAMBIA1304/*AS1* vector). Plates were then incubated at 28˚C in a growth chamber with a 16-h florescent light (100 µmol/m2/sec) and 8-h dark cycle. Explants were regularly subcultured to new MS medium supplemented with 250 mg.L^−1^ cefotaxime and suitable antibiotic (75 mg.L^−1^ Hygromycin and 50 mg.L^−1^ Kanamycin) every two weeks. The single shoot regenerated from inoculated explants was excised from the calli and transferred onto the MS basal medium supplemented with 100 mg.L^−1^ cefotaxime. Rooted plantlets were then transferred into pots containing 70% peatmoss; 30% perlite and grown in above-mentioned conditions.

### CAPS analysis

#### DNA extraction and PCR

Total DNA was extracted from fresh petal of putative transgenic plants with CTAB method as previously described [44]. For PCR analysis, p1 and p2 specific primers were used for *4’CGT* and *AS1* genes (Supplementary Table S2). PCR was performed using GoTaq® Green Master Mix (Promega, Madison, WI, USA) in a T100 thermal cycle (Bio-Rad), with initial denaturation at 95 ºC for 5 min, followed by 35 cycles at 95 ºC for 45 s, 58 ºC for 30 s and 72 ºC for 2 min, and a final extension step at 72 ºC for 10 min. The analysis of products was performed via 1% agarose gel electrophoresis and sequencing (Sanger Sequencing method, ABI 3730 XL).

### Southern blot analysis

For Southern blot analysis, genomic DNA of petals was treated with 10 µg L^−1^ RNase for 4 h at 25 °C, followed by phenol-chloroform extraction and ethanol precipitation. Representative probes were prepared with digoxigenin, by used DIG DNA labeling and detection kit. 30 µg DNA of each leaves sample was cut with *NcoI* enzyme for *AS1* and *BamHI* enzyme for *4CGT* (Thermo Fisher) by incubating for 16 h at 37 °C. The digested products were separated by 1% agarose gel, denatured in 1.5 M NaCl, 0.5 M NaOH for 30 min each, and transferred to hybridization membrane (GeneScreen, DuPont, Boston, MA, USA). Hybridization was performed at 65 °C with digoxigenin-labeled probe (Amersham Rediprim II, GE Healthcare, Pittsburgh, PA, USA). After 20 h of hybridization, the membrane was washed twice in 2× SSC at room temperature for 15 min each, twice in 2× SSC, 1% SDS at 65 °C for 30 min each, and finally once in 0.1× SSC at room temperature for 30 min. The membrane was exposed to an imaging plate at room temperature and the signal was detected by a phosphor imager (Typhoon FLA 7000, GE Healthcare, Pittsburgh, PA, USA).

### Quantitative realtime PCR (qRTPCR) for the expression analysis of *4’CGT* and *AS1* genes

For real-time quantitative PCR, total RNA was extracted from African violet white (non-transformation), transgenic A. violet, and *A. majus* petals all treated with *DNase* I, as previously described by wang et al. [45]. Next, cDNA was synthesized from total RNA (100 ng) using the Superscript III First-Strand Synthesis System (Invitrogen, Thermo Fisher Scientific, Waltham, MA, USA) and oligo (dT) 20. The transcript levels of *4’CGT* and *AS1* were analyzed via RT-qPCR (Applied Biosystems 7900HT Fast Real-Time PCR System) using Power SYBRTM Green PCR Master Mix (Applied Biosystems, Thermo Fisher Scientific) according to the manufacturer’s instructions. Transcript levels were calculated based on ∆∆Cq (formerly ∆∆Ct) method [46] using *Actin* (accession number: AB596843.1) gene as references. Statistical significance of the differential expression levels was assessed as independent experiments (with mean centering and autoscaling) [47]. The Tukey–Kramer test at the 1% level was used for the analysis of aurone biosynthesis-related genes. The results are presented as the standardized mean of SE.

### Light Microscopy analysis

The morphology of regenerates transgenic and non-transgenic petals were examined under a VH-Z75 light microscope. Petals were cut into 5 × 5 mm pieces and embedded in 4% agarose. Thin sections (thickness, 100 mm) were cut with a Micro Slicer DTK-1000 (D.S.K.). Sections were examined using a VH-Z75 light microscope (Keyence).

### Aureusidin 6-*O* glucoside identification by HPLC-DAD-MS^n^

The African violet white, transgenic African violet, and *A. majus* petals (12 mm) were evaluated separately. Aurone analyses were performed using an Agilent HPLC series 1200 equipped with ChemStation software, a degasser, quaternary pumps, autosampler with chiller, column oven, and diode array detector. The guard column operated at a temperature of 35 C. The mobile phase consisted of 0.1% TFA⁄water (eluent A) and 90% acetonitrile in 0.1% TFA⁄water (eluent B) at a flow of 0.8 mL⁄min using the following gradient program: 20% B (0–3 min); 20–60% B (3–20 min); 60% B isocratic (20–27 min); 60–90% B washing step (27–30 min); and equilibration for 10 min. The total run time was 40 min. The injection volume for all samples was 10 L. Specific wavelengths were monitored separately at 400 nm for aurone and 360 nm for flavones. Additionally, UV⁄Vis spectra were recorded at 520 nm for anthocyanins. The HPLC system was coupled online to a Bruker (Bremen, Germany) ion trap mass spectrometer fitted with an ESI source. Data acquisition and processing were performed using Bruker software. The mass spectrometer was operated in positive ion mode and auto MSn with a scan range from m⁄z 100 to 1000. HPLC-grade acetonitrile, water, trifluoroacetic acid (TFA), naringenin, and chalcone standards were purchased from Sigma (St. Louis, MO). All standards were prepared as stock solutions at 10 mg⁄mL in methanol and diluted in water except for chalcone, which was prepared in 50% methanol. UV external standard calibration was also used to obtain calibration curves of cyanidin-3-O-glucose, naringenin-7-O-rutinoside, and chalcone, which were used to quantify anthocyanins, flavones, and chalcones, respectively. UV external calibration of maritime was employed for the quantitation of *AOG*.

## Supplementary Information

**Additional file 1:** **Table S1.** Genes used for RT-qPCR in African violet white (wild type), transgenic African violet and *A. majus* (Yellow). **Table S2.** List of primers used in the study. 

## Data Availability

The data sets supporting the conclusions of this article are included within the article and its additional files.

## Abbreviations

4'CGT: Chalcone 4'-*O*-glucosyltransferase
AS1: Aureusidin synthase
ATMT: Agrobacteriumtumefaciens-mediated transformation
THC: 2',4',6',4 tetrahydroxychalcone
CHS: Chalcone synthase
CHI: Chalcone isomerase
AOG: Aureusidin-6-*O*-glucose
F3H: Flavanone 3-hydroxylase
DFR: Dihydroflavonol 4-reductase
