# Supplementary material for: Genetic engineering of novel yellow color african violet (Saintpaulia ionantha) produced by accumulation of Aureusidin 6-O-glucoside
Source: Biol Proced Online. 2022 Feb 9;24:3. doi: 10.1186/s12575-022-00164-0 (PMC8903692; doi:10.1186/s12575-022-00164-0)
Supplement: Supplementary file 1 — Additional file 1: Table S1. Genes used for RT-qPCR in African violet white (wild type), transgenic African violet and A. majus (Yellow). Table S2. List of primers used in the study. [file 12575_2022_164_MOESM1_ESM.docx]

| **Supplementary Table 1.** Genes used for RT-qPCR in African violet white (wild type), transgenic African violet and *A. majus* (Yellow) | | | | | |
| --- | --- | --- | --- | --- | --- |
| **Gene** | **Genebank ID** | **Product length (bp)** | **Melting temperature (˚C)** | **Amplification efficiency (%)** | **Correlation coefficient R^2^** |
| *4'CGT* | AB198665 | 120 | 60 | 99.86 % | 0.977 |
| *AS1* | AB044884 | 111 | 60 | 98.71 % | 0.996 |
| Actin | AB596843.1 | 86 | 60 | 99.83 % | 0.984 |

| **Supplementary Table 2.** List of primers used in the study | | | | |
| --- | --- | --- | --- | --- |
| **No** | **Forward Primer** | **5'-Sequence-3'** | **Reverse Primer** | **5'-Sequence-3'** |
| 1 | BamHI-4'CGT-FW | GGGGATCCATGGGAGAAGAATACAAGAAA | SacI- 4'CGT-RV | GGGAGCTCTTAACGAGTGACCGAGTTGAT |
| 2 | NcoI- AS1-FW | GGCCATGGATGTTCAAAAATCCTAATATC | BstEII -AS1-RV | GGGGTTACCTTAGCCATCAAGCTCAATCTT |
| 3 | 4'CGT-FW | CCGAACCCCACGACACTAAA | 4'CGT-RV | GTGCTGAGAACGCTCCTCTT |
| 4 | AS1-FW | TTATCGTGACGAGGCCGAAG | AS1-RV | ATTTTACGTGCCCGTGGTCT |
| 5 | 4'CGT probe-FW | ATGGGAGAAGAATACAAGAAAAC | 4'CGT probe-RV | TCTTACGATAAAACAAACTCA |
| 6 | AS1-probe-FW | AATTATTTCCCAATGTTCAAAAAT | AS1-probe-RV | TGGAGCTTTAGGTTTGTGAAA |
| 7 | ACTIN-FW | TTGATTCTGGTGACGGGGTG | ACTIN-RV | AGCAAGATCCAACCGCAGAA |
